# α-Blocker Use in Hemodialysis: The Japan Dialysis Outcomes and Practice Patterns Study

**DOI:** 10.1016/j.xkme.2023.100698

**Published:** 2023-07-04

**Authors:** Ken Iseri, Chisato Miyakoshi, Nobuhiko Joki, Yoshihiro Onishi, Shingo Fukuma, Hirokazu Honda, Kazuhiko Tsuruya

**Affiliations:** 1 Division of Nephrology, Department of Medicine, Showa University School of Medicine, Tokyo, Japan; 2 Department of Research Support, Center for Clinical Research and Innovation, Kobe City Medical Center General Hospital, Hyogo, Japan; 3 Division of Nephrology, Toho University Ohashi Medical Center, Tokyo, Japan; 4 Institute for Health Outcomes and Process Evaluation Research (iHope International), Kyoto, Japan; 5 Human Health Sciences, Graduate School of Medicine, Kyoto University, Kyoto, Japan; 6 Department of Nephrology, Nara Medical University, Nara, Japan

**Keywords:** α-blocker, hypertension, fall, fracture, dialysis

## Abstract

**Rationale & Objective:**

Despite α-blockers’ use for hypertension as add-on therapy in patients treated with hemodialysis, scant information is available on their association, particularly with safety, in these patients.

**Study Design:**

Prospective cohort study.

**Setting & Participants:**

patients treated with hemodialysis and receiving antihypertensive agents in the Japan Dialysis Outcomes and Practice Patterns Study, phases 4-6, were analyzed.

**Exposure:**

Primary exposure was the prescription of α-blocking antihypertensive agents at baseline.

**Outcomes:**

Incident fractures, falls, and all-cause mortality.

**Analytical Approach:**

Multivariable Cox and modified Poisson regression analysis.

**Results:**

Of 5,149 patients treated with hemodialysis (mean age, 65 years; 68% men) receiving antihypertensive drugs, 717 (14%) received α-blocking agents. During a mean follow-up period of 2.0 years, 247 fractures, 525 falls, and 498 deaths occurred. Multivariable analysis showed no significant association of α-blocker use and increased risk of fractures (hazard ratio [HR], 0.92 [95% confidence interval {CI}, 0.61-1.38]), falls (HR, 0.94 [95% CI, 0.74-1.20]), or all-cause deaths (HR, 0.87 [95% CI, 0.64-1.20]) compared with α-blocker nonuse. α-Blocker use was, however, significantly associated with a decreased risk of all-cause mortality in the subgroup analysis, for example, patients who were older (HR, 0.71 [95% CI, 0.51-0.99]), were women (HR, 0.68 [95% CI, 0.48-0.95]), or reported a history of cardiovascular disease (HR, 0.67 [95% CI, 0.48-0.95]) or a predialysis blood pressure of ≥140 mm Hg (HR, 0.69 [95% CI, 0.49-0.98]).

**Limitations:**

Selection bias cannot be ruled out given the prevalent user analysis.

**Conclusions:**

No significant association between α-blocker use and the risk of worse safety-related outcomes was seen, indicating that clinicians may safely prescribe α-blockers to patients receiving hemodialysis who require blood pressure lowering.

**Plain-Language Summary:**

α-Blockers have been generally reserved for use as add-on therapy for resistant or refractory hypertension. However, little is known about the safety of α-blockers in patients treated by hemodialysis. We analyzed 5,149 patients receiving hemodialysis in Japan who were receiving antihypertensive drugs from the Japan Dialysis Outcomes and Practice Patterns Study. The results showed no significant increase in the risk of fractures, falls, or deaths for patients using α-blockers compared with those who did not, suggesting that α-blockers may be safely prescribed for patients receiving hemodialysis who need to lower their blood pressure.

## Introduction

Hypertension caused by volume expansion, activation of the renin-angiotensin system, or arteriosclerosis is highly prevalent among patients receiving dialysis and associated with increased mortality.^1^, ^2^, ^3^, ^4^ Achieving optimal dry weight after assessment of volume status, ensuring adequate duration of dialysis, restricting dietary sodium, and use of antihypertensive medications are measures that lead toward optimal blood pressure. The achievement of desired blood pressure using pharmacological agents is associated with decreased risks of cardiovascular mortality and all-cause death.^5^ Thus, maintaining optimal blood pressure control is important for better clinical outcomes.

α-Blockers are among the antihypertensive drugs that decrease blood pressure by reducing peripheral vascular resistance.^6^ Some previous studies, however, have accumulated evidence regarding the associations of α-blockers with an increased risk of orthostatic hypotension, falls, and fractures.^7^, ^8^, ^9^, ^10^ Conversely, other studies have shown limited efficacy of α-blockers in improving clinical outcomes, such as cardiovascular events and all-cause mortality.^10^^,^^11^ In light of these safety and efficacy concerns, renin-angiotensin system inhibitors (RASis) and calcium-channel blockers, which offer cardiovascular protection with fewer serious safety concerns, are used as first-line drugs for hypertension management in both patients treated with dialysis and the general population.^12^, ^13^, ^14^ α-Blockers have been generally reserved for use as add-on therapy for resistant or refractory hypertension.^14^

It is well established that patients receiving hemodialysis have poorer outcomes compared with nondialysis patients with chronic kidney disease, because of their elevated inflammatory cytokines, frailty, malnutrition, and progression of atherosclerosis.^15^^,^^16^ Furthermore, intradialytic hypotension resulting from loss of fluid with each session of dialysis therapy is often seen in clinical practice, possibly resulting in such multiple complications as falls, fractures, cardiovascular disease, and all-cause death.^17^, ^18^, ^19^ Most clinical studies in this field exclude patients treated with dialysis and patients with severe kidney failure. It has been well described that treating with α-blockers shows a significant association with a higher risk of hypotension-related events^9^^,^^10^; however, data on the safety of α-blockers in patients treated with dialysis are scant. Because adverse events that are confirmed in the general population may be even more serious in patients receiving hemodialysis, clinicians are often hesitant to prescribe α-blockers in clinical practice, even when the blood pressure exceeds the target range.

The aim of this study was to investigate the safety—incidence of fractures, all-cause mortality, and falling—of α-blockers in the population receiving hemodialysis using data from the Japan Dialysis Outcomes and Practice Patterns Study (J-DOPPS) cohort. We also explored differences in associations among α-blockers in subgroup populations.

## Methods

### Study Design and Participants

The Dialysis Outcomes and Practice Patterns Study (DOPPS) is a prospective cohort study conducted worldwide to clarify the association between hemodialysis practice and patient outcomes, such as mortality, hospitalization, health-related quality of life, and vascular access. J-DOPPS, a part of DOPPS, gathers data from randomly selected adult patients receiving hemodialysis at >60 randomly selected dialysis facilities in Japan.

We used data from J-DOPPS phases 4 (2009-2012), 5 (2021-2015), and 6 (2015-2018). We excluded patients who received no antihypertensive agent at baseline. All patients provided written informed consent, and the study protocol was approved by the ethics committee of Kyoto university graduate school and faculty of medicine (approval number: R1301). Details of DOPPS sampling and study methods have been described previously.^20^

### Definition of Exposure

The primary exposure was the prescription of α-blocking antihypertensive agents at baseline (retrieved from medical records just before the start of the follow-up). The drugs included were doxazosin mesylate, bunazosin hydrochloride, prazosin hydrochloride, and urapidil hydrochloride. In sensitivity analyses, a time-dependent prescription of α-blockers was used as the exposure.

### Outcomes

Our primary outcome was incident fractures, defined as emergency department visits or hospitalizations caused by fractures. Data from the time of patient enrollment to the first such episode were retrieved through medical records by each facility. Kidney transplantation, transfer to another dialysis unit, or the end of the observation period of each J-DOPPS phase, whichever came first, was considered a censoring event.

We also examined all-cause mortality and falls as secondary outcomes. Data on mortality were retrieved through medical records; data on falls were collected from patient-reported questionnaires asking “have you fallen in the past 12 months (yes or no)? A fall, in this case, is defined as an accidental fall to the ground, floor, or other lower place. It does not mean falling from a high place.” (These data were collected only in 2016 and 2017.)

### Covariates

The data collected as adjusting covariates are as follows: age, sex, primary cause of kidney failure, hemodialysis vintage (years), smoking status, body mass index (<18.0 kg/m^2^, 18.0 to <22.0 kg/m^2^, 22.0 to <25.0 kg/m^2^, and ≥25.0 kg/m^2^), comorbidities (cerebrovascular disease, peripheral vascular disease, diabetes, lung disease, cancer, neurologic disease, and cardiac vascular diseases), prescribed antihypertensive medications (RASi, calcium-channel blocker, ®-blocker, and diuretic), serum albumin (<3.0 g/dL, 3.0 to <3.5 g/dL, and≥3.5 g/dL), hemoglobin (<10.0 g/dL, 10.0 to <12.0 g/dL, and ≥12.0 g/dL), serum phosphorus (<3.5 mg/dL, 3.5-6.0 mg/dL, and >6.0 mg/dL), intact parathyroid hormone (iPTH) (<60 pg/mL, 60-240 pg/mL, and >240 pg/mL), serum C-reactive protein (<0.3 mg/dL, 0.3 to <1.0 mg/dL, 1.0 to <3.0 mg/dL, and ≥3.0 mg/dL), daily urine volume (≤200 mL/d and >200 mL/d), fluid removal (<4%, 4 to <6%, and ≥6%), and predialysis systolic blood pressure (<110 mm Hg, 110 to <130 mm Hg, 130 to <150 mm Hg, ≥150 mm Hg), Kt/V (<1.2, 1.2 to <1.6, ≥1.6). All of these variables were used in each adjusted model in the study.

### Statistical Analysis

The baseline characteristics were summarized using means (standard deviation) or medians (25th and 75th percentiles) for continuous variables and percentages for categorical variables. Differences in patient characteristics between groups were summarized using standardized mean differences.

For the time-to-event outcomes, we estimated crude and adjusted hazard ratios (HRs) and 95% confidence intervals (CIs) using Cox regression models; all of the previously mentioned covariates were adjusted as clinically relevant confounding.

In the analysis of falling events, the risk ratio and 95% CI were estimated using modified Poisson regression models.^21^ We did not use logistic regression models because the incidence proportion of falling events was expected to be >10%. Since multiple events could be counted for each patient, we used a generalized estimating equation approach with an exchangeable correlation structure to incorporate within-patient correlations.

Missing data were imputed using the multivariate imputation by chained equation (mice) algorithm with the predictive mean matching method for all types of variables. The results for 50 imputed datasets were combined using Rubin’s rule.

As a sensitivity analysis, we conducted propensity score analyses with inverse probability of treatment weighting (IPTW). Within each imputed dataset, the propensity score was estimated using a logistic regression model, including all covariates listed above as the regressors. We estimated the average treatment effects using the stabilized weight trimmed at the 99th percentile.

To confirm the robustness of the results from the baseline Cox regressions, we did a second sensitivity analysis: we analyzed the associations of time-dependent exposures with outcomes of incident fractures and all-cause mortality using a marginal structural model (MSM).^22^^,^^23^ In that model, the unit period of exposure was set at 4 months, which was the data collection interval of DOPPS. The time-varying weights were calculated from the inverse of the probability of having the history of the use of α-blockers that a patient actually had during each 4-month period of observation. These probabilities were predicted from a pooled logistic regression model with the time-dependent use of α-blockers as the dependent variable and the time-dependent predictors as the independent variables. The predictors were all covariates listed above.

We also conducted subgroup analyses to investigate the effect heterogeneity of α-blocking antihypertensive agents. We estimated adjusted HRs or risk ratios using the models mentioned previously. The *P* value adjustment for multiplicity was not performed because this was an exploratory analysis.

All statistical analyses were performed using the R software program, version 4.1.0 (R foundation for statistical computing). Two-tailed *P* values of <0.05 were considered statistically significant.

## Results

### Patient Flow

A total of 7,128 patients participated in J-DOPPS phases 4, 5, and 6. After excluding those who were not prescribed any antihypertensive agent, 5,149 patients were included in the primary analysis (Table S1). In the analysis of fall outcomes, 1,723 responses from 1,047 patients were included.

### Patient Characteristics

The baseline characteristics of 5,149 patients are summarized in Table 1. The mean age was 65.4 ± 12.2 years, and 68% of the patients were men. Among patients who were prescribed >1 antihypertensive agent, 14% (717/5,149) received α-blocking agents. Patients receiving α-blocking agents were likely to be prescribed a larger number of antihypertensive medications, and RASi and calcium-channel blocker prescriptions were more frequent than prescriptions without α-blocking agents. Baseline characteristics of 1,047 patients in the analysis of fall outcomes are shown in Table S2, which gives the same findings as above.

**Table 1:** Baseline Characteristics of Study Patients

Characteristics	All Patients (N = 5,149) (%)		Use of α-Blockers		
		Missing Data (%)	No (n = 4,432) (%)	Yes (n = 717) (%)	Standardized Mean Difference
Age	65.4 (12.2)	3 (0.1)	65.7 (12.1)	63.8 (12.3)	0.15
Male	3,498 (68)	3 (0.1)	3,008 (68)	490 (68)	0.01
Primary cause of kidney failure	—	260 (5)	—	—	0.22
Diabetic nephropathy	2,068 (42)	—	1,727 (41)	341 (50)	—
Chronic glomerulonephritis	1,488 (30)	—	1,299 (31)	189 (28)	—
Nephrosclerosis	394 (8)	—	339 (8)	55 (8)	—
Polycystic kidney disease	257 (5)	—	229 (5)	28 (4)	—
Others	682 (14)	—	616 (15)	66 (10)	—
Hemodialysis vintage (y)	2.8 (0.4-7.6)	18 (0.3)	2.8 (0.4-7.6)	2.7 (0.4-7.5)	0.07
Residual urine volume of >200mL/d	941 (30)	2,048 (40)	816 (30)	125 (30)	0.00
Body mass index (kg/m^2^)	21.7 (3.7)	360 (7)	21.7 (3.6)	22.1 (4.0)	0.13
Predialysis systolic blood pressure (mm Hg)	152 (23)	148 (3)	151 (23)	156 (22)	0.22
Current smoker	676 (21)	1,918 (37)	574 (21)	102 (20)	0.11
Fluid removal rate (%)	4.0 (2.7)	129 (3)	4.0 (2.8)	4.1 (1.8)	0.11
Single pool (Kt/V)	1.3 (0.3)	842 (16)	1.3 (0.3)	1.3 (0.3)	0.12
Comorbidities					
Diabetes	2,378 (46)	0	1,997 (45)	381 (53)	0.16
Cardiovascular disease	2,919 (57)	0	2,540 (57)	379 (53)	0.09
Cerebrovascular disease	751 (15)	0	651 (15)	100 (14)	0.02
Peripheral vascular disease	759 (15)	0	643 (15)	116 (16)	0.05
Cancer	604 (12)	0	542 (12)	62 (9)	0.12
Neurologic disease	365 (7)	0	325 (7)	40 (6)	0.07
Lung disease	174 (3)	0	148 (3)	26 (4)	0.02
Laboratory					
Hemoglobin (g/dL)	10.6 (1.3)	42 (1)	10.6 (1.3)	10.5 (1.2)	0.02
Albumin (g/dL)	3.6 (0.5)	180 (3)	3.6 (0.5)	3.6 (0.4)	0.05
Phosphorus (mg/dL)	5.3 (1.4)	53 (1)	5.3 (1.4)	5.4 (1.5)	0.03
Intact parathyroid hormone (pg/mL)	137 (76-225)	1,176 (23)	136 (75-225)	141 (80-228)	0.02
C-reactive protein (mg/dL)	0.1 (0.1-0.4)	1,663 (32)	0.1 (0.1-0.4)	0.1 (0.1-0.3)	0.01
Antihypertensive medications					
Renin-angiotensin system inhibitors	3,386 (66)	0	2,808 (63)	578 (81)	0.39
Calcium-channel blocker	3,114 (60)	0	2,566 (58)	548 (76)	0.40
®-Blocker	1,737 (34)	0	1,484 (33)	253 (35)	0.04
Diuretics	2,384 (46)	0	2,067 (47)	317 (44)	0.05
Number of antihypertensives	2.4 (1.3)	0	2.2 (1.1)	3.8 (1.3)	1.30

*Note*: Values are mean ± SD, median (interquartile range), or number (proportion).

### Primary Outcome

**Figure 1:**
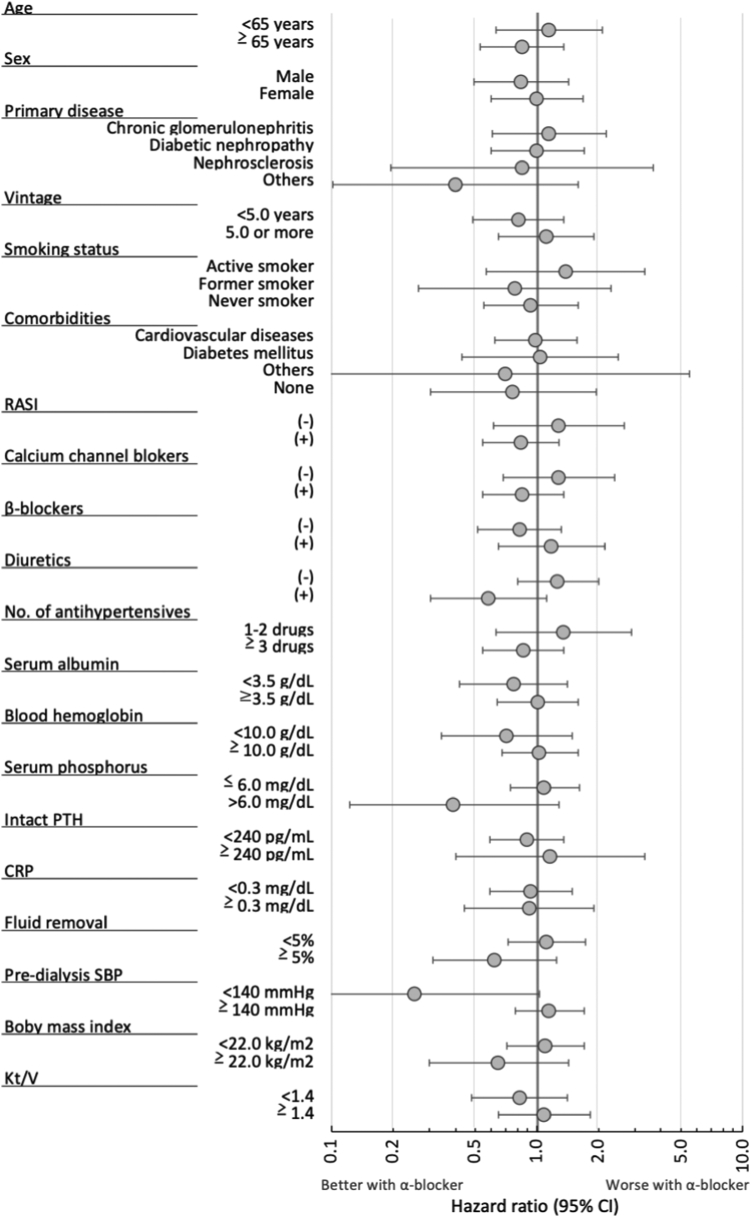
Adjusted hazard ratios and 95% confidence intervals for the incidence of fractures associated with the use of α-blocking agents stratified by patient characteristics. There are no subgroups with a statistically significant difference. RASi, renin-angiotensin system inhibitor; PTH, parathyroid hormone; CRP, C-reactive protein; SBP, systolic blood pressure.

During a mean follow-up period of 2.0 ± 1.0 years, emergency department visits or hospitalizations were recorded because fractures were reported among 33 of the 717 patients (4.6%) with α-blocking agents and 214 of 4,432 patients (4.8%) without α-blocking agents. The estimated HR for the first episode of fracture is summarized in the left column of Table 2. The multivariable regression model results revealed that the use of α-blocking agents was not associated with a greater incidence of fractures, and this was consistent with the result of sensitivity analysis using IPTW. The second sensitivity analysis using MSM gave similar estimates (HR, 1.16 [95% CI, 0.78-1.71; *P* = 0.47]). In the subgroup analyses, no heterogeneity across subgroups was noted (Fig 1).

**Table 2:** Estimated Hazard Ratios for 3 Outcomes Associated With the Use of α-Blocking Agents

	Primary Outcome	Secondary Outcomes	
	First Episode of Fracture	All-Cause Mortality	Annual Incidence of Fall
Number of patients/measurements	5,149	5,149	1,723
Number of outcome events	247	498	525
Analytical model	Cox regression	Cox regression	Modified Poisson regression
Outcome measure	Hazard ratio	Hazard ratio	Risk ratio
Estimates (95% CI)	—	0.74 (0.55-0.98); *P* = 0.03	—
Crude analysis	0.93 (0.64-1.34); *P* = 0.68	—	0.96 (0.77-1.19); *P* = 0.70
Adjusted analysis^a^	1.08 (0.65-1.79); *P* = 0.76	0.79 (0.54-1.16); *P* = 0.23	1.10 (0.82-1.47); *P* = 0.54
Sensitivity analysis (IPTW^a^)	0.92 (0.61-1.38); *P* = 0.67	0.87 (0.64-1.20); *P* = 0.40	0.94 (0.74-1.20); *P* = 0.61

*Note:* Hazard ratios or risk ratios associated with the use of α-blocking agents versus nonuse.

Abbreviations: CI, confidence interval; IPTW, inverse probability of treatment weighting.

a Each separate analysis included all covariates indicated in the text (methods section).

### Secondary Outcomes

#### All-Cause Mortality

**Figure 2:**
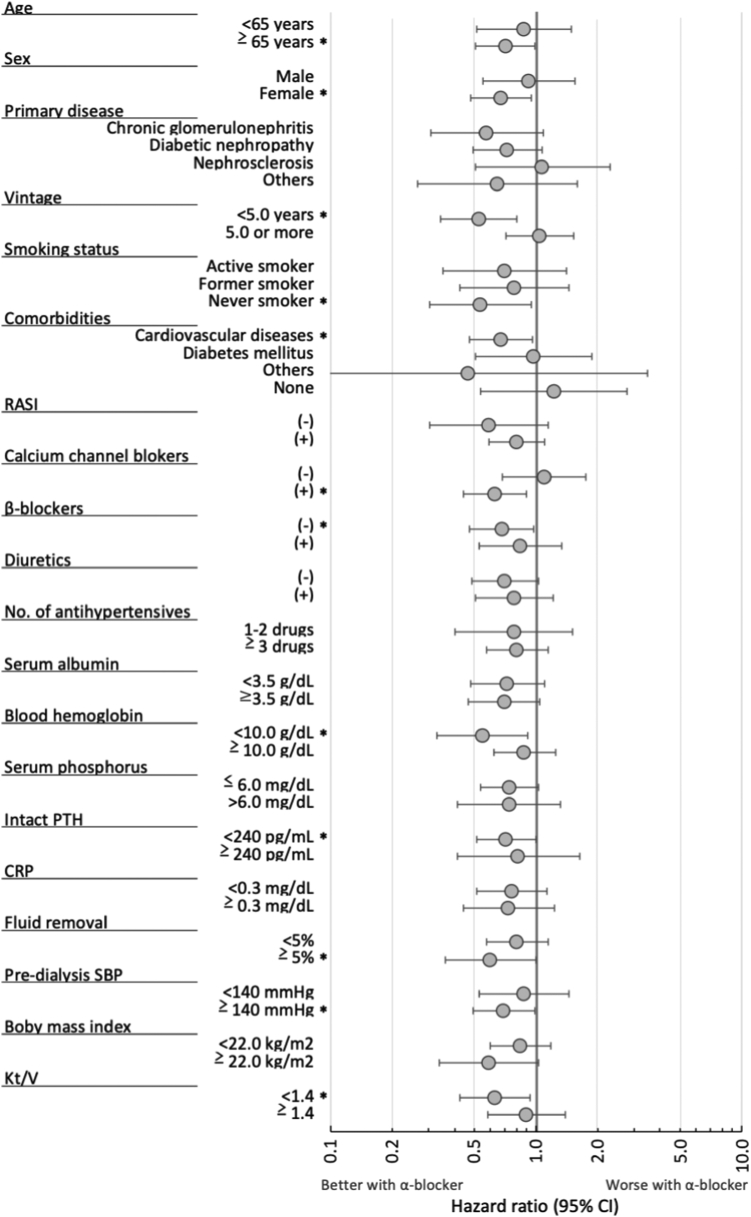
Adjusted hazard ratios and 95% confidence intervals for all-cause mortality associated with the use of α-blocking agents stratified by patient characteristics. ∗*P* < 0.05. RASi, renin-angiotensin system inhibitor; PTH, parathyroid hormone; CRP, C-reactive protein; SBP, systolic blood pressure.

During the follow-up period, 7.5% (54/717) of those prescribed α-blocking agents died; 10.0% (444/4,432) of those not prescribed α-blocking agents died. The HR for all-cause mortality is shown in the second column of Table 2. Analyses using a multivariable regression model revealed no significant differences between groups, nor did the analysis using IPTW reveal any significant differences. The second sensitivity analysis using MSM showed a significant difference (HR, 0.61 [95% CI, 0.43-0.86; *P* ] 0.004]); however, the finding was the same in that the use of α-blocking agents was not associated with increased mortality. In the subgroup analyses (Fig 2), lower HR for deaths associated with the use of α-blocking agents were noted among the following subgroups: age >65 years (HR, 0.71 [95% CI, 0.51-0.99]), female sex (HR, 0.68 [95% CI, 0.48-0.95]), never smoker (HR, 0.53 [95% CI, 0.30-0.94]), vintage period of <5 years (HR, 0.53 [95% CI, 0.34-0.81]), cardiovascular disease comorbidity (HR, 0.67 [95% CI, 0.48-0.95]), receiving calcium-channel blockers (HR, 0.63 [95% CI, 0.44-0.90]), not receiving ®-blocking agents (HR, 0.68 [95% CI, 0.48-0.98]), hemoglobin level of <10 g/dL (HR, 0.55 [95% CI, 0.33-0.91]), serum iPTH level of <240 pg/mL (HR, 0.71 [95% CI, 0.51-0.99]), predialysis systolic pressure of ≥140 mm Hg (HR, 0.69 [95% CI, 0.49-0.98]), fluid removal of ≥5% (HR, 0.60 [95% CI, 0.36-0.99]), and Kt/V <1.4 (HR, 0.63 [95% CI, 0.43-0.93]).

#### Falls

Among patients treated with α-blocking agents, 69 of 238 (29.0%) of responses reported no fall over the last 12 months, very similar to the responses from patients not treated with α-blocking agents (456/1,485; 30.7%). The estimated risk ratios for falls are described in the third column of Table 2. There was no significant difference in the incidence of falls between groups in the analyses using the multivariable regression model, or IPTW. Subgroup analyses revealed that use of α-blocking agents was not associated with the incidence of falls among any subpopulation (Fig S1).

## Discussion

Associations of α-blockers with incidents of fractures, all-cause death, and falling in patients receiving hemodialysis remain unexamined despite the use of α-blockers in clinical practice as add-on therapy for resistant or refractory hypertension. In this study using J-DOPPS data, we investigated the associations, particularly with safety, of α-blockers in patients treated with hemodialysis. Contrary to expectations, based on multivariable analysis, our study showed α-blocker use not to be significantly associated with an increased risk of fractures and falls. Results were robust in multiple subgroups and in a sensitivity analysis using IPTW. The second sensitivity analysis using time-dependent models showed similar results: α-blocker use was not associated with an increased risk of fractures or all-cause mortality. Subgroup analysis enabled us to identify certain subgroups with α-blocker use that showed a favorable effect on all-cause mortality, but not total populations.

Hypertension is common in patients receiving hemodialysis; 75% of all patients treated with dialysis in Japan have hypertension^13^; this is concordant with the present study’s finding that more than 72% of the participants enrolled in J-DOPPS 4-6 had received α-blockers or other antihypertensive drugs and their mean predialysis blood pressure level exceeded 150 mm Hg. The predialysis blood pressure levels were significantly higher in α-blocker users than in α-blocker non-users, suggesting that α-blockers were commonly used in patients treated with hemodialysis with resistant hypertension.

The best way to measure blood pressure, diagnostic procedures for hypertension, and blood pressure target ranges for patients receiving hemodialysis continue to be debated worldwide.^24^, ^25^, ^26^ Currently, Japanese Society for Dialysis Therapy guidelines recommend that the predialysis blood pressure (before the first dialysis procedure of the week) should be <140/90 mm Hg.^13^ The U-shaped curve relationship between blood pressure levels and outcomes in patients treated with hemodialysis is well documented and differs from that in the general population.^24^, ^25^, ^26^ Moreover, a rapid decrease in blood pressure during dialysis therapy and orthostatic hypotension—falling blood pressure on standing—are also associated with a worsening prognosis.^27^^,^^28^ The prevalence of orthostatic hypotension and serious decline in systolic blood pressure are more pronounced in patients with poor kidney function.^29^ In patients receiving hemodialysis, orthostatic hypotension is more commonly seen in post-dialysis than in predialysis therapy and may be caused by the rapid removal of fluid volume during dialysis, in addition to other common risk factors such as older age, diabetes, and medications.^30^

In this study, we found surprisingly that α-blocker use reported no significant association with an increased risk of fractures and falls. These findings were moreover not heterogeneous across subgroups: they applied even in patients with risk factors related to falls and fractures, such as advanced age, a low systolic blood pressure of <140 mm Hg, anemia, low BMI, and large fluid removal volume. Our findings regarding concern for safety diverged from previous studies focusing on general populations, which showed that α-blocker use was associated with an increased risk of falls and fracture.^7^^,^^9^ One possible explanation for the discrepancy between the population treated with hemodialysis and the general population is the greater provider-patient visit frequency in populations treated with hemodialysis than in the general population. Face-to-face meetings with nephrologists 3 times per week may permit avoidance of undesirable events through achievement of optimal blood pressure management. Similarly, a population-based retrospective cohort study from Canada also showed that α-blocker use was significantly associated with a lower risk (HR, 0.75; 95% CI, 0.58-0.97) of hypotension among those with a lower estimated glomerular filtration rate (eGFR < 30 mL/min/1.73 m^2^), and no significant association with a higher risk of syncope, falls, or fractures, whereas patients with higher eGFRs (60-89 mL/min/1.73 m^2^) had an increased risk of hypotension and syncope.^11^ In addition to superior safety, the Canadian study also showed an association of α-blocker use with a decreased risk of all-cause death in patients with lower eGFR (<60 mL/min/1.73 m^2^), but not in those with higher eGFR (≥60 mL/min/1.73 m^2^). Another contributory factor is that a greater provider-patient visit frequency may be required in patients with a low eGFR than in those with a high eGFR owing to frequent adjustments in such drugs as erythropoiesis-stimulating agents and diuretics.

Our subgroup analysis also found that patients with a history of cardiovascular disease were a subgroup associated with a decreased risk of all-cause mortality; this was also supported by the Canadian study showing that α-blocker use in patients with heart failure was associated with a lower incidence of heart-failure-related readmission and a reduction in total mortality, this reduction being greater in those receiving higher doses of α-blocker.^31^ In our study focusing on patients treated with hemodialysis, we also found that certain other subpopulations’ α-blocker use is significantly associated with a decrease in all-cause mortality risk, whereas no such significant association has been found in the total population receiving hemodialysis. We could not fully explain why this favorable effect was found only in specific populations treated with hemodialysis but not in total populations. However, recent studies have shown that α-blockers may prevent cardiac remodeling and the development and progression of heart failure and have protective benefits against hyperinflammation and cytokine storm syndrome.^32^, ^33^, ^34^ Furthermore, research is required to investigate if and how α-blockers may produce favorable outcomes in specific subgroups of patients receiving dialysis.

The strengths of the present study include the use of J-DOPPS, a nationally representative prospective cohort study of randomly selected patients treated with hemodialysis from facilities across Japan. Our results are likely generalizable to Japanese clinical practice. In addition, J-DOPPS is a rich source of data on dialysis therapy, such as predialysis blood pressure, fluid removal rates, and Kt/V, which are known to be associated with clinical outcomes. Regarding outcomes, we used a repeated questionnaire that captured fall incidents regardless of severity; if we had relied solely on diagnostic codes from hospitalizations or emergency room visits, clinically significant falls not resulting in hospitalizations or emergency room visits would have been missed. Thus, our study was able to capture outcomes regardless of their severity.

Some limitations should be considered when interpreting the results. First, as in any observational study, we cannot conclude causality. Second, many confounders, such as muscle strength, physical activity, bone histology findings, bone mineral density, health status such as frailty index, alcohol consumption, intradialytic hypotension, history of orthostatic hypotension, falls, and previous doxazosin exposure which conceivably may affect the risk of fractures and falls—are unaccounted for. Also, we have no information about the severity of falls, cause of falls, and loss of consciousness from a fall. Third, because of lacking drug information including adherence to α-blocker therapy, we used the primary intention-to-treat approach in the present study. This allowed for complete follow-up and estimation of the overall association of treatment with α-blocker use, but it might also have resulted in exposure misclassification. However, the time-dependent analyses showed consistent results, suggesting the robustness of our findings.

In conclusion, in patients treated with hemodialysis receiving antihypertensive agents, α-blocker use compared with α-blocker nonuse was not significantly associated with any increased risk of fractures and falls. Clinicians may safely prescribe α-blockers when considering additional antihypertensive therapies for patients undergoing hemodialysis. Our findings merit further investigation of their external validity.
