# Supplementary material for: α-Blocker Use in Hemodialysis: The Japan Dialysis Outcomes and Practice Patterns Study
Source: Kidney Med. 2023 Jul 4;5(9):100698. doi: 10.1016/j.xkme.2023.100698 (PMC10470217; doi:10.1016/j.xkme.2023.100698)
Supplement: Supplementary File (PDF) — Fig S1; Tables S1 and S2. [file mmc1.pdf]

Figure S1 Adjusted risk ratios and 95% confidence intervals (CI) for fall associated with the use of alpha-blocking agents stratified by patient characteristics. There are no subgroups with a statistically significant difference. Abbreviations: RASi, renin-angiotensin system inhibitors; PTH, parathyroid hormone; CRP, C-reactive protein; SBP, systolic blood pressure.

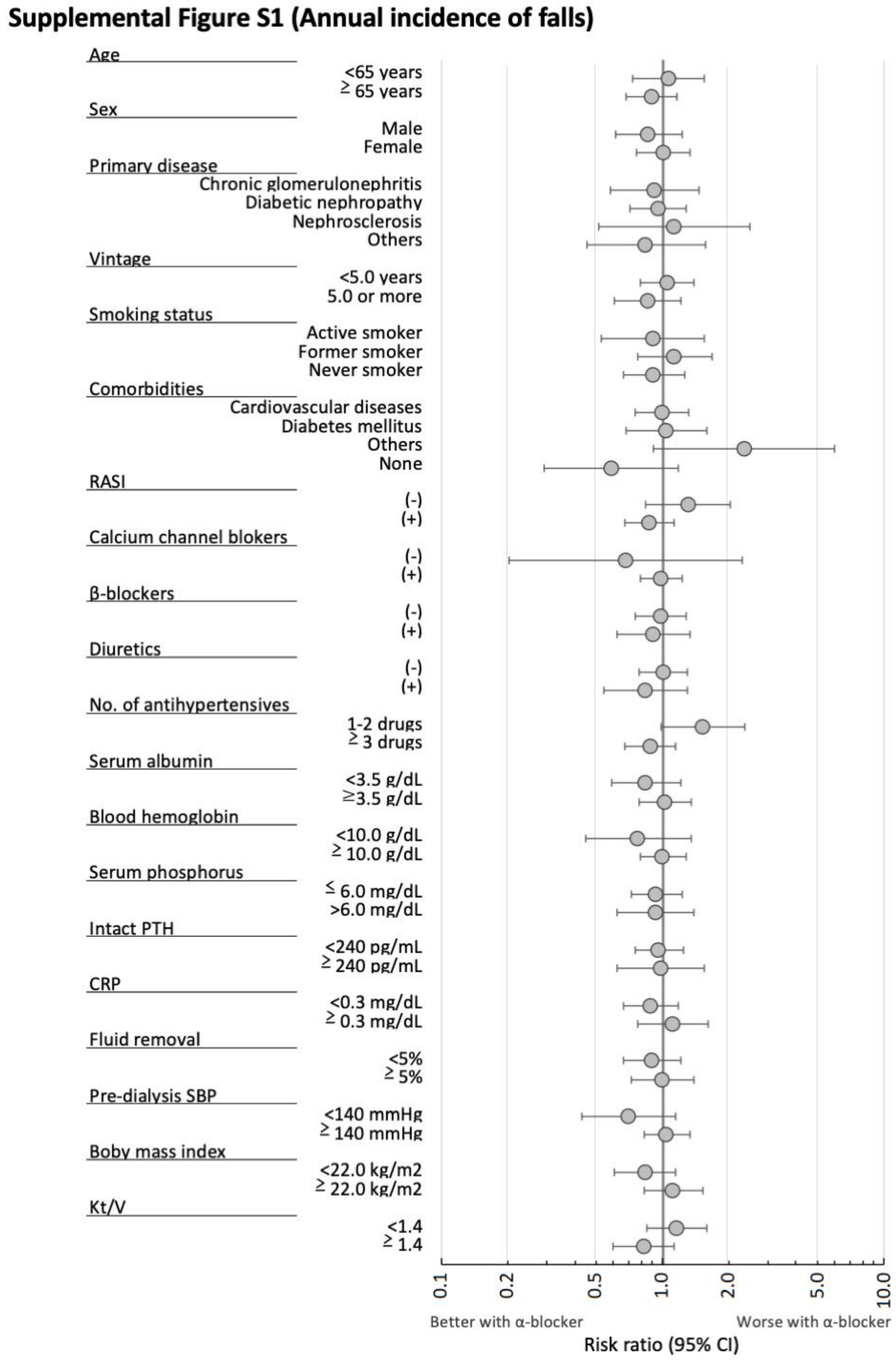

Table S1. Selection of the patients for analysis

(A) ANALYSES FOR ALL-CAUSE MORTALITY, CARDIOVASCULAR MORTALITY, AND FRACTURES

	Number
Participants of J-DOPPS 4, 5, and 6 with follow-up data	7128
EXCLUDED:	
No use of antihypertensive drugs	-1979
**Subjects analyzed**	**5149**

(B) ANALYSES FOR FALLS
(USING DATA FROM PATIENT-REPORTED OUTCOMES)

	Number
Participants of J-DOPPS 6 with follow-up data	2341
EXCLUDED:	
Information of falls not available	-921
No use of antihypertensive drugs	-373
**Subjects analyzed**	**1047***

* A total of 1723 responses were collected for two questionnaires during the follow-up.

Table S2. Baseline characteristics of patients for analysis of fall events

Items	All patients (N=1047)	Missing data	Use of alpha-blockers		
			No (N=903)	Yes (N=144)	Standardized mean difference
Age	65.0 (11.5)	0	65.0 (11.6)	64.8 (11.5)	0.02
Male sex	711 (68%)	0	620 (69%)	91 (63%)	0.12
Primary cause of end-stage kidney disease		33 (3%)			0.16
Diabetic nephropathy	418 (41%)		350 (40%)	68 (48%)	
Chronic glomerulonephritis	306 (30%)		268 (31%)	38 (27%)	
Nephrosclerosis	86 (8%)		75 (9%)	11 (8%)	
Polycystic kidney disease	67 (7%)		58 (7%)	9 (6%)	
Others	137 (14%)		121 (14%)	16 (11%)	
Hemodialysis vintage (years)	4.3 [1.9, 9.4]	0	4.4 [2.0, 9.5]	4.1 [1.7, 9.4]	0.11
Residual urine volume of >200mL/day	188 (21%)	155 (15%)	165 (21%)	23 (19%)	0.05
Body mass index (kg/m^2)	22.1 (3.9)	41 (4%)	22.1 (3.8)	22.2 (4.1)	0.02
Pre-dialysis systolic blood pressure (mmHg)	152 (22)	10 (1%)	152 (22)	157 (22)	0.23
Current smoker	146 (16%)	142 (14%)	122 (15%)	24 (21%)	0.16
Fluid removal rate (%)	4.3 (1.5)	11 (1%)	4.2 (1.5)	4.6 (1.5)	0.23
Single pool Kt/V	1.5 (0.3)	115 (11%)	1.5 (0.3)	1.5 (0.3)	0.08
Comobidities					
Diabetes	484 (46%)	0	404 (45%)	80 (56%)	0.22
Cardiovascular disease	622 (59%)	0	552 (61%)	70 (49%)	0.25
Cerebrovascular disease	187 (18%)	0	163 (18%)	24 (17%)	0.04
Peripheral vascular disease	129 (12%)	0	114 (13%)	15 (10%)	0.07
Cancer	129 (12%)	0	115 (13%)	14 (10%)	0.10
Neurologic disease	44 (4%)	0	38 (4%)	6 (4%)	0.00
Lung disease	30 (3%)	0	27 (3%)	3 (2%)	0.06
Laboratory					
Hemoglobin (g/dL)	11.0 (1.2)	2 (0%)	11.0 (1.2)	10.8 (1.1)	0.15
Albumin (g/dL)	3.7 (0.4)	11 (1%)	3.7 (0.4)	3.6 (0.4)	0.22
Phosphorus (mg/dL)	5.3 (1.3)	3 (0%)	5.3 (1.3)	5.3 (1.4)	0.05
Intact parathyroid hormone (pg/mL)	127 [71, 210]	114 (11%)	126 [71, 214]	137 [74, 205]	0.00
C-reactive protein (mg/dL)	0.1 [0.1, 0.4]	236 (23%)	0.1 [0.1, 0.4]	0.1 [0.1, 0.3]	0.04
Antihypertensive medications					
Renin-angiotensin system inhibitors	692 (66%)	0	574 (64%)	118 (82%)	0.42
Calcium channel blocker	733 (70%)	0	600 (66%)	133 (92%)	0.68
β-blocker	411 (39%)	0	356 (39%)	55 (38%)	0.03
Diuretics	339 (32%)	0	298 (33%)	41 (28%)	0.10
Number of antihypertensives	2.5 (1.3)	0	2.2 (1.2)	3.8 (1.3)	1.26

Values are mean (standard deviation), median [interquartile range], or number (proportion).
